# Inhibition of phosphoinositide 3-kinase activity attenuates neutrophilic airway inflammation and inhibits pyrin domain-containing 3 inflammasome activation in an ovalbumin-lipopolysaccharide-induced asthma murine model

**DOI:** 10.1007/s11033-024-09360-5

**Published:** 2024-05-29

**Authors:** Qun Li, Guiyun Jiang, Yunxiang Lv

**Affiliations:** 1 Department of Pulmonary and Critical Care Medicine, The Second Affiliated Hospital of Bengbu Medical University, Anhui, China; 2 Department of Clinical Laboratory, The First Affiliated Hospital of Bengbu Medical University, Bengbu, 233000 Anhui China; 3 Department of Pulmonary and Critical Care MedicineAnhui Clinical and Preclinical Key Laboratory of Respiratory DiseaseMolecular Diagnosis Center, The First Affiliated Hospital of Bengbu Medical University, Bengbu, 233000 Anhui China

**Keywords:** Interleukin-17, LY294002, Neutrophilic airway inflammation, NLRP3, Phosphoinositide 3-kinase

## Abstract

**Background:**

Existing investigations suggest that the blockade of phosphoinositide 3-kinase (PI3K) activity contributes to inflammatory solution in allergic asthma, but whether this inhibition directly attenuates neutrophilic airway inflammation in vivo is still unclear. We explored the pharmacological effects of LY294002, a specific inhibitor of PI3K on the progression of neutrophilic airway inflammation and investigated the underlying mechanism.

**Methods and results:**

Female C57BL/6 mice were intranasally sensitized with ovalbumin (OVA) together with lipopolysaccharide (LPS) on days 0 and 6, and challenged with OVA on days 14–17 to establish a neutrophilic airway disease model. In the challenge phase, a subset of mice was treated intratracheally with LY294002. We found that treatment of LY294002 attenuates clinic symptoms of inflammatory mice. Histological studies showed that LY294002 significantly inhibited inflammatory cell infiltration and mucus production. The treatment also significantly inhibited OVA-LPS induced increases in inflammatory cell counts, especially neutrophil counts, and IL-17 levels in bronchoalveolar lavage fluid (BALF). LY294002 treated mice exhibited significantly increased IL-10 levels in BALF compared to the untreated mice. In addition, LY294002 reduced the plasma concentrations of IL-6 and IL-17. The anti-inflammatory effects of LY29402 were correlated with the downregulation of NLRP3 inflammasome.

**Conclusions:**

Our findings suggested that LY294002 as a potential pharmacological target for neutrophilic airway inflammation.

**Supplementary Information:**

The online version contains supplementary material available at 10.1007/s11033-024-09360-5.

## Introduction

Asthma is a heterogeneous chronic inflammatory disease [1]. In general, eosinophilic asthma can be well treated by inhalation of corticosteroids, whereas patients display with neutrophilic asthma are usual unresponsive to corticosteroid treatment, resulting in poor disease control and frequent exacerbations [2]. Despite multiple novel targets on neutrophilic asthma have been developed, the lack of clinical improvements limited the therapeutic value of these targets in severe asthma [3]. Therefore, it is urgent to identify potential new therapeutic targets for treatment of neutrophilic asthma.

Type 17 helper T cells (Th17) that mainly produces interleukin 17 (IL-17) have been involved in the pathogenesis of neutrophilic asthma [4]. After being activated by Th17-derived mediators including endotoxin and IL‐1 receptor accessory protein (IL1RAP), the airway epithelial cells further release Interleukin (IL)‐33, neutrophil elastase and matrix metalloproteases for the recruitment of the neutrophils [5, 6]. Studies have shown that the number of neutrophils in sputum, bronchial alveolar lavage fluid (BALF) and bronchial biopsies were higher in patients with neutrophilic asthma than those of healthy patients or patients with eosinophilic asthma [7, 8]. Accordingly, increased levels of IL-17 in BALF and IL-6 and IL-17 in blood have been reported in patients with severe neutrophilic asthma [3, 8].

Phosphoinositide 3-kinases (PI3K) are a family of intracellular signaling proteins involved in various cellular processes including survival, proliferation, differentiation and migration, as well as involved in the pathogenesis of various diseases, such as allergy, cancer and inflammation [9]. Treatment of LY294002, a specific inhibitor of PI3K, significantly reduced allergic airway inflammation in an ovalbumin (OVA)-induced murine asthma model [10]. Moreover, the recent study showed that LY294002 also significantly inhibited IL-25-induced lung tissue eosinophilia, mucus production, collagen deposition, smooth muscle hypertrophy and angiogenesis [11]. Similarly, administration of IC87114, a selective PI3K δ inhibitor, attenuates allergic airway inflammation in asthma mice [12, 13]. Furthermore, IC87114 also significantly reduced the increase in IL-17 protein and mRNA expression in allergic mice [13].

The nucleotide-binding oligomerization domain-like receptor (NLR) family, pyrin domain-containing 3 (NLRP3) inflammasome is the most widely characterized and involved in inflammatory diseases [14]. Recent findings have reported that *Chlamydia* and *Haemophilus* infections increased expression levels of NLRP3, caspase-1 and IL-1β in OVA-induced severe asthma murine model [15]. Furthermore, there was significantly elevated gene expression of NLRP3, caspase-1 and IL-1β in patients with neutrophilic asthma [16].

In the present study, we hypothesized that LY294002 might be able to attenuate neutrophilic airway inflammation. We thus evaluated the effects of LY294002 administration and investigated the mechanism in a mouse model of neutrophilic airway inflammation.

## Methods

### Animals

Female C57BL/6 mice (6–8 weeks) were obtained from the Shanghai Laboratory Animal Center and were housed under specific pathogen-free conditions at the Department of Laboratory Animals Center. All of the animal experiments were strictly conducted under protocols approved by the Institutional Animal Care and Use Committee of the University of Science and Technology of China. Mice were randomly distributed into four groups (n = 8 each): the control group (Con), the OVA + LPS group (OVA + LPS), the control + LY294002 group (Con + LY), and the OVA + LPS + LY294002 group (OVA + LPS + LY).

### Experimental protocols and treatments

The neutrophilic airway inflammation mouse model was established as previously reported with minor modification [17]. Briefly, to induce sensitization, mice were sensitized by oropharyngeal administrations of 100 μg OVA (Chicken Egg OVA, Grade V; Sigma) and 0.1 μg LPS (St. Louis, MO, USA, Sigma) in 50 μL saline on days 0 and 6. The OVA + LPS sensitized mice with or without LY294002 (80 μg/50 μL in 0.1% dimethyl sulfoxide (DMSO) in saline, San Diego, CA, USA) were given intratracheally 2 h before each 1% OVA aerosol challenge on days 14–17 for 30 min. Control mice were saline-sensitized and challenged with nebulized saline solution containing 0.1% DMSO. Shortness of breath, wheezing, and sneezing are common clinical signs of asthma. Because spotting these symptoms in mice can be difficult, they show signs like scratching, tickling, and rapid breathing. Therefore, the behavior of challenged mice nasal scratching was evaluated last 10 min after the final challenge. The scoring was as follows: mice who scratched their noses 0–2 times scored 0 points, 3–5 times scored 1 point, 6–8 times scored 2 points, and nine times or more scored 3 points as mentioned in a previous study [18]. All of the mice were killed 24 h after the final challenge. The left lung was harvested and subjected to bronchoalveolar lavage and subsequent differential cell counting and ELISA analysis, and the right lung was used for histopathological analysis and further Western blotting assays.

### Histological analysis

Lung tissues were fixed in 4% paraformaldehyde. The paraffin-embedded tissues were sectioned (5-μm thickness) and stained with hematoxylin/eosin (H&E) or periodic acid-Schiff (PAS) to measure inflammatory cellular infiltration or mucus production, respectively. The quantitative analysis of peri-bronchial inflammation in H&E-stained lung slices and the quantification of mucus production was accomplished by assessing the number of PAS^+^ cells in the airway as in our previous studies [18].

### Bronchoalveolar lavage fluid (BALF) and cellular analysis

BALF was collected by lavage of the left lung with 0.5 mL of ice-cold PBS. The fluid was centrifuged at 700 g at 4 °C for 5 min. The cell pellets harvested from the BALF were resuspended in 200 μL PBS. Total cells were counted using a hemocytometer, and differential cells number was identified by staining cytospins of BALF samples with Wright Stain solution (Sigma). At least 200 cells were counted for each mouse. The supernatants were stored at – 80 °C for ELISA analysis.

### Enzyme-linked immunosorbent assay (ELISA)

The production of IL-4, IL-10, IL-13 and IL-17 in BALF, and IL-6 and IL-17 in plasma were measured by ELISA using specific kits (Cusabio, Wuhan, China) according to the protocols from the manufacturer. The specificities of IL-4, IL-6, IL-10, IL-13 and IL-17 were 0.39 pg/mL, 7.8 pg/mL, 1.56 pg/mL, 0.8 pg/mL and 11.75 pg/mL respectively.

### Western blotting

The lung tissues were homogenized using a homogenizer with RIPA buffer containing a protease inhibitor cocktail (Roche, Indianapolis, IN, USA) and phosphatase inhibitor PhosSTOP (Roche). Total protein was separated by SDS-PAGE and transferred to PVDF membranes (Millipore, Billerica, MA, USA). Membranes were blocked in 5% nonfat milk and then processed with primary antibodies: anti-p-Akt/Akt (1:1000), anti-NLRP3 (1:1000) and anti-ASC (1:1000) (cell signaling technology Inc., Beverly, MA, USA), anti-caspase-1 (1:500), anti-IL-18 (1:500) and anti-IL-1β (1:500) (Santa Cruz Biotechnology, CA, USA), and anti-GAPDH (1:2000) (KANGCHEN Biotech, Shanghai, China). All of the membranes were subsequently incubated with HRP-conjugated anti-rabbit IgG (Promega, Madison, WI, USA), polyclonal rabbit anti-mouse IgG (Dako, Copenhagen, Denmark), and all of the blots were detected via enhanced chemiluminescence (ECL; Thermo Scientific). For quantitative analysis, the intensity of protein bands was determined using Image J 1.38 × software (NIH, Bethesda, MD, USA).

### Statistical analysis

Statistical analysis was performed using SPSS 16.0. All of the data were expressed as the mean ± SD of triplicate samples and were representative of at least three separate experiments. Independent-sample *t* tests or one-way ANOVA with a post hoc Bonferroni’s test was used for all of the statistical analysis. Values of *P* < 0.05 were considered significant.

## Results

### LY294002 attenuates clinical symptoms of inflammatory mice and pathologic features of allergic mice

**Fig. 1:**
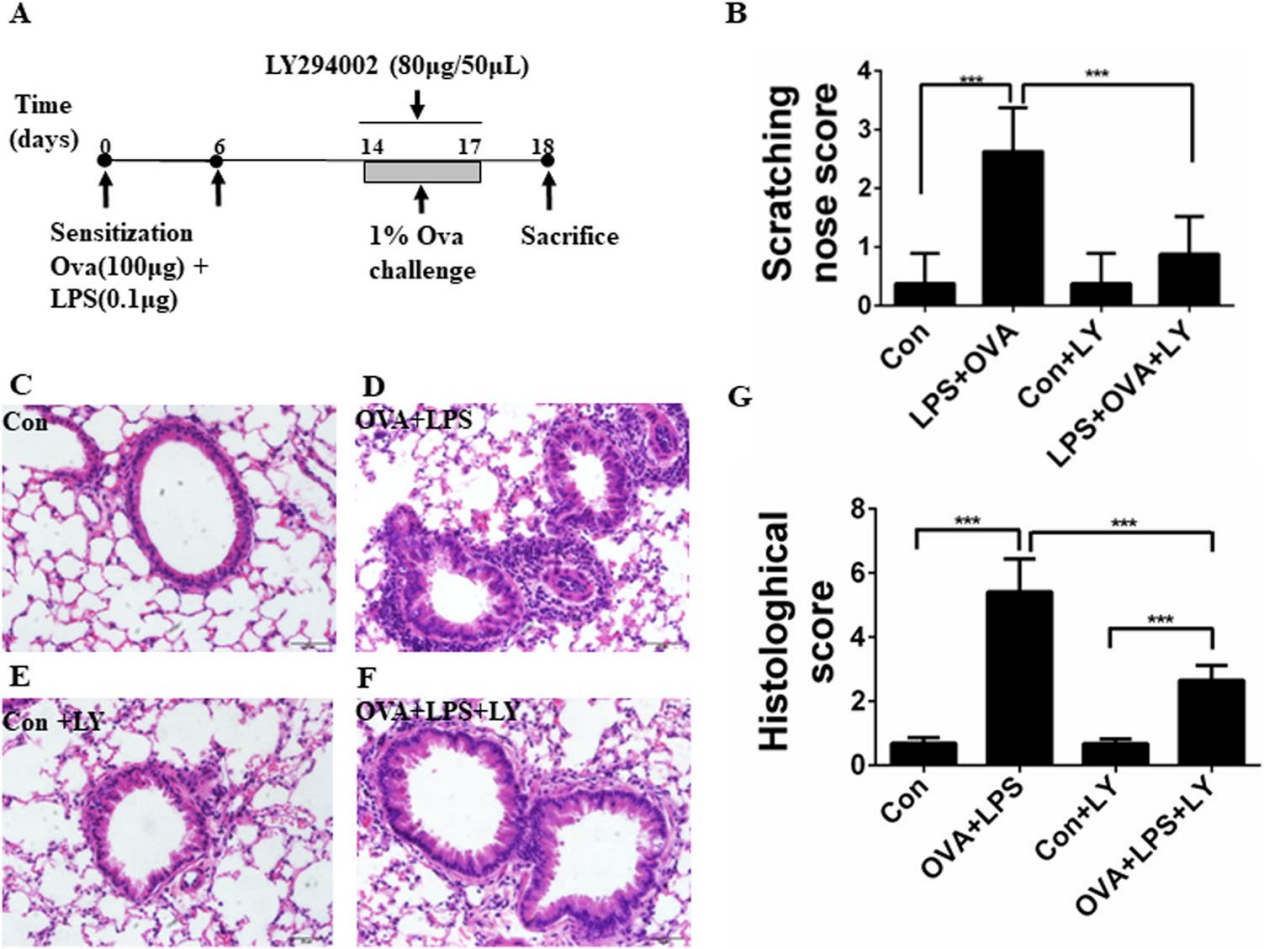
Treatment of LY294002 ameliorates clinical symptom and pathologic features of OVA-induced allergic mice. **A** Experimental protocol of establishment of neutrophilic airway disease model in C57BL/6 mice. **B** The scratching nose scores of mice in four different groups. **C**–**F** Representative H&E-staining of lung sections from the control group (**C**), the OVA + LPS group (**D**), the Con + LY group (**E**), the OVA + LPS + LY group (**F**), Bars: 50 μm. **G** Histological scoring of lung inflammatory of C57BL/6 mice. Data were expressed as the means ± SD of 8 mice per group. ***P < 0.001

C57BL/6 mice were sensitized and challenged as illustrated in Fig. 1A. In the aerosol challenge phase, mice in the OVA + LPS group manifested significantly elevated nasal scratching scores compare to the mice in the control group. Mice in the OVA + LPS + LY group manifested the significantly decreased nasal scratching scores compare to the mice in the OVA + LPS group. The nasal scratching scores of mice in the control group and Con + LY group were very low (Fig. 1B). Histological staining revealed that more inflammatory cells were recruited into the peribronchial regions in the lungs of the OVA + LPS group mice compared with the control group mice (Fig. 1, C, D, G ). The challenged mice administered with LY294002 showed a significant reduction of inflammatory cell recruitment in the peri-bronchial regions (Fig. 1D, E, G).

### LY294002 reduces the invasion of inflammatory cells in BALF

**Fig. 2:**
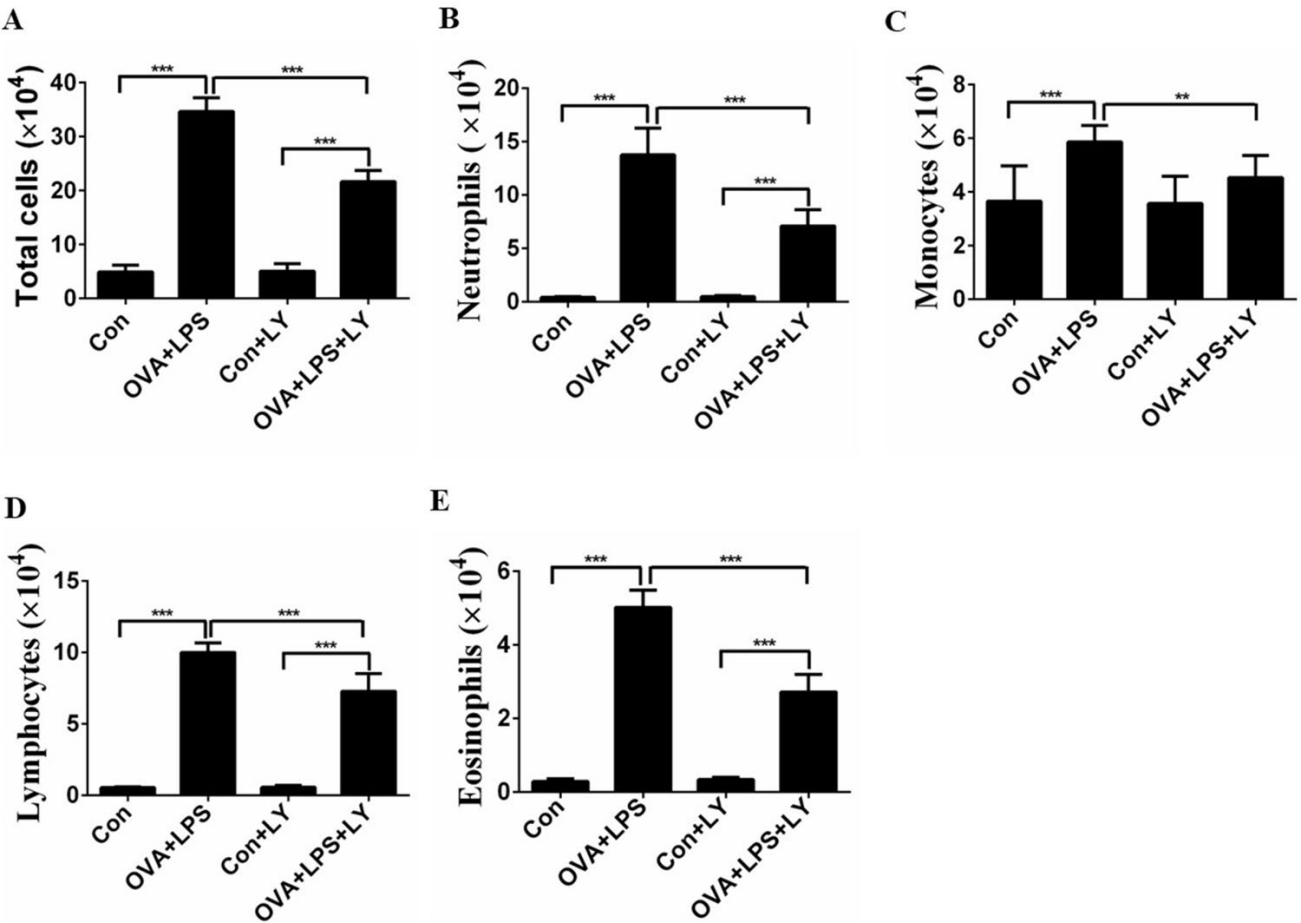
Treatment of LY294002 reduces the invasion of inflammatory cells in BALF. **A**–**E** Total and differential cell counts in BALF. Data were expressed as the means ± SD of 8 mice per group. Compared to the OVA + LPS group, **P < 0.01, ***P < 0.001

We counted the number of inflammatory cells in the BALF. The numbers of total cells, neutrophils, eosinophils, lymphocytes, and monocytes of the allergic mice were significantly elevated compared with those of the control mice (Fig. 2A–E). Treatment with LY294002 produced a significant decrease in released inflammatory cells, especially neutrophils (Fig. 2A–E).

### LY294002 affects the production of inflammatory cytokines in BALF

**Fig. 3:**
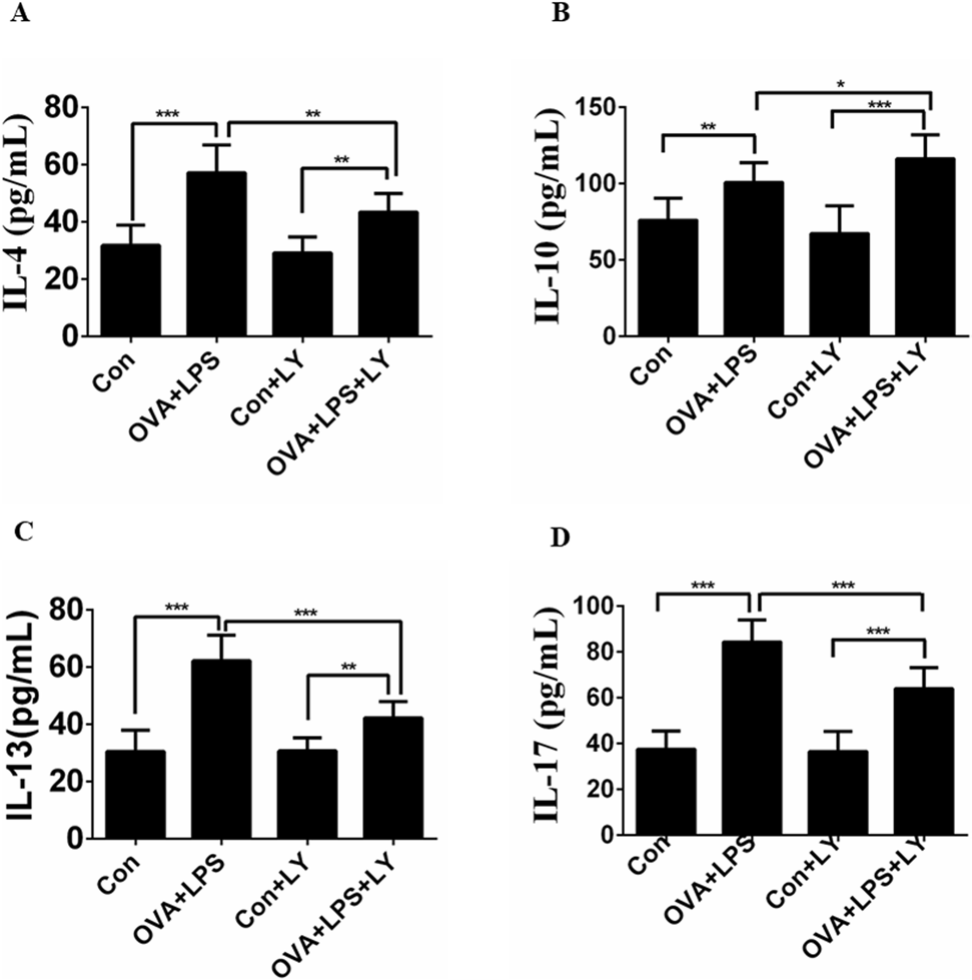
Administration of LY294002 affects the production of IL-4, IL-10, IL-13 and IL-17 in BALF. **A** Secretion levels of IL-4 in BALF. **B** Secretion levels of IL-10 in BALF. **C** Secretion levels of IL-13 in BALF. **D** Secretion levels of IL-17 in BALF. Data were expressed as the means ± SD of 8 mice per group. Compared to the OVA + LPS group, *P < 0.05, **P < 0.01, ***P < 0.001

ELISA analysis showed that the production of IL-4, IL-10, IL-13 and IL-17 in the BALF of the allergic mice was also significantly increased compared with that of the control mice (Fig. 3). Concurrently, treatment with LY294002 significantly reduced the secretion of IL-4 (Fig. 3A), IL-13 (Fig. 3C) and IL-17 (Fig. 3D) in the BALF. However, allergic mice treated with LY294002 significantly increased the production of IL-10 in the BALF (Fig. 3B).

### LY294002 reduces the plasma levels of IL-6 and IL-17 in inflammatory mice

**Fig. 4:**
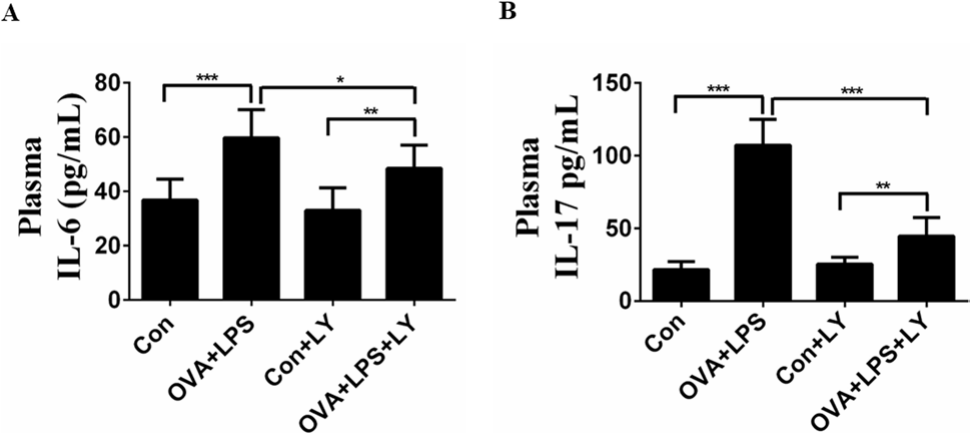
Administration of LY294002 reduces the plasma levels of IL-6 and IL-17 in neutrophilic airway inflammatory mice. **A** Plasma levels of IL-6 by ELISA. **B** Plasma levels of IL-17 by ELISA. Data were expressed as the means ± SD of 8 mice per group. Compared to OVA group, *P < 0.05, ***P < 0.001

We assessed the systemic immune response by measuring plasma IL-6 and IL-17 levels using ELISA. The plasma levels of IL-6 and IL-17 in the OVA + LPS group mice were significantly higher than those of in the control group mice (Fig. 4A, B). Moreover, LY294002 treatment markedly decreased the plasma levels of IL-6 and IL-17 relative to the untreated mice (Fig. 4A, B).

### LY294002 decreased mucus secretion and the NLRP3 inflammasome in allergic lungs

**Fig. 5:**
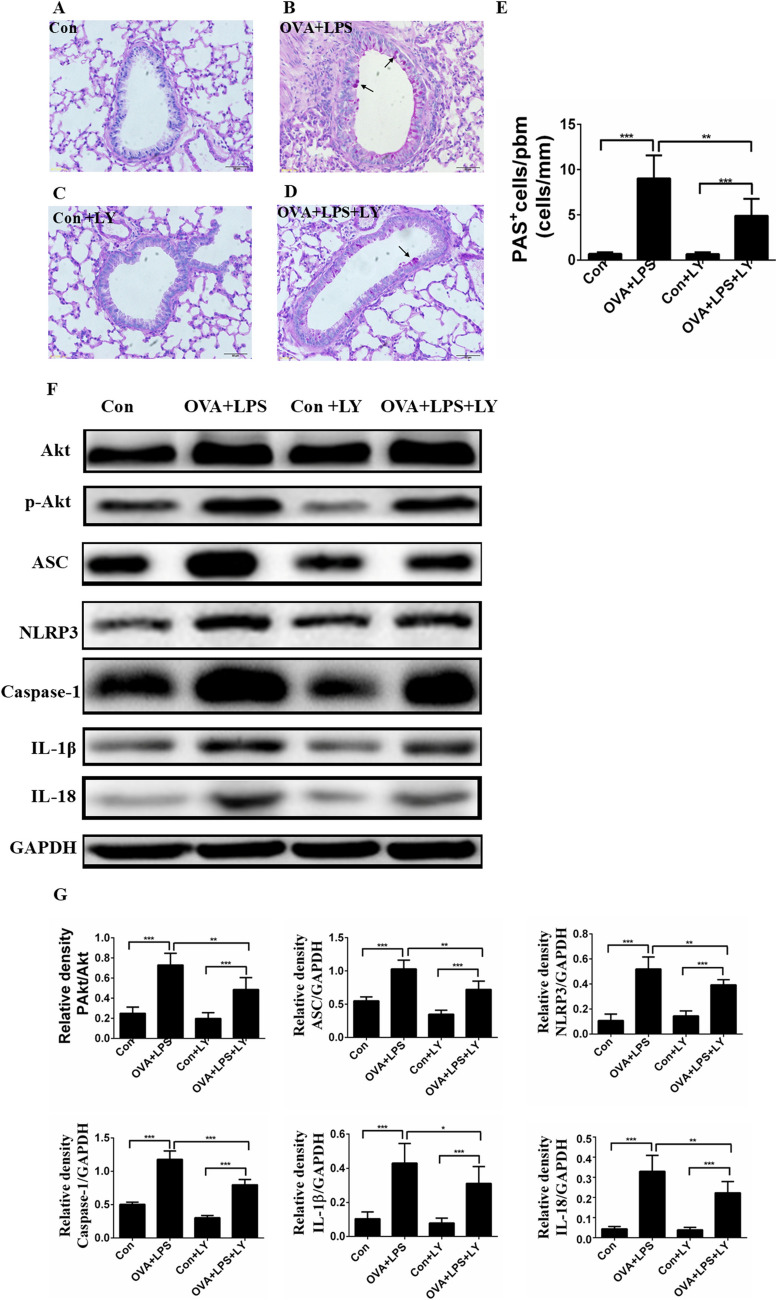
LY294002 decreased mucus production and the NLRP3 inflammasome expression in allergic lungs. **A**–**D**) Representative periodic acid-Schiff (PAS) staining of lung sections from mice in the control group (**A**), the OVA + LPS group (**B**), the Con + LY group (**C**), the OVA + LPS + LY group (**D**). **E** Quantification of PAS.^+^ cells in the airway of mice. **F** Measurement of p-Akt, ASC, NLRP3, caspase-1, IL-1β and IL-18 protein expression in mice by Western blotting. **G** The relative protein levels of p-Akt, ASC, NLRP3, caspase-1 IL-1β and IL-18 in the lung. Gels were representative of three different experiments. Data were expressed as the means ± SD of 8 mice per group. *P < 0.05, **P < 0.01, ***P < 0.001

We measured mucus secretion and cell hyperplasia by PAS staining as described in Methods. PAS^+^ cells surrounding the airway were significantly increased in the OVA + LPS group mice compared to that of control mice (Fig. 5A, B, E). Furthermore, LY294002 treatment significantly reduced the PAS^+^ cells surrounding the airway relative to the untreated mice (Fig. 5B, D, E)*.* To explore its possible underlying mechanisms, we analyzed the phosphorylation levels of p-Akt and NLRP3 inflammasome related proteins in the lungs by Western-blotting. OVA + LPS group mice exhibited significantly increased protein phosphorylation of p-Akt compared to control mice (Fig. 5F, G). The expression levels of p-Akt were significantly decreased by treatment with LY294002 relative to that in the untreated group (Fig. 5F, G). Similarly, the expression levels of ASC, NLRP3, caspase-1, IL-1β and IL-18 of mice in the OVA + LPS group were significantly increased relative to that in the Control group (Fig. 5F, G). Furthermore, challenged mice treated with LY294002 showed significantly reduced ASC, NLRP3, caspase-1, IL-1β and IL-18 expression compared to untreated mice (Fig. 5F, G).

## Discussion

In the present study, we assessed the effects of pharmacological treatment with PI3K inhibitor LY294002 on an OVA-LPS-induced murine model of neutrophilic airway inflammation. Applying histological, biochemical, and molecular analyses, we found that LY294002 promoted neutrophilic airway inflammatory resolution. Moreover, LY294002 also reduced the plasma concentrations of IL-6 and IL-17. The anti-inflammatory effects in airways were correlated with the downregulation of NLRP3 inflammasome. Our findings suggested that LY294002 as a potential pharmacological target for neutrophilic airway inflammation.

Severe asthma is characterized by episodic dysphoria, neutrophilic inflammation, either in the presence or absence of Th2-induced eosinophilic inflammation [2]. In the present study, to quantify these clinical symptoms in the mice is too difficult to acquire. Thus, in our study we assessed the asthmatic behavior through measuring nasal scratching scores according to a previous study [18]. We found that LY294002 significantly reduced nasal scratching scores of OVA-LPS-challenged mice. Moreover, neutrophil numbers and IL-17 levels in BALF were significantly increased in these challenged mice compared to that in control mice, suggesting that the neutrophilic airway inflammation mouse model was established as previously reported [19, 20]. These results were further confirmed neutrophil numbers in sputum, BALF and bronchial biopsies were higher in neutrophilic asthma than those of control mice.

LY294002, a relatively specific PI3K inhibitor, can inhibit the kinase activity by competing with the ATP binding [21]. Evidence has suggested that blockade of PI3K signaling pathway may be a potential therapeutic strategy for asthma and rhinovirus-induced exacerbations [22]. In an OVA-induced murine asthma model, treatment of LY294002 significantly reduced allergic airway inflammation [10]. Moreover, the recent study showed that LY294002 also significantly inhibited IL-25-induced lung tissue eosinophilia, mucus production, collagen deposition, smooth muscle hypertrophy and angiogenesis [11]. Here, in line with previous studies, we also found the elevated levels of IL-4 and IL-13 were significantly reduced in through treatment of LY294002 in OVA-LPS challenged mice [10, 11, 13]. An inspiring study reported that treatment of LY294002 restored histone deacetylase 2 and steroid sensitivity in a mouse model of severe, steroid-insensitive allergic airway disease [23]. A recent study showed that inhalation of the prodrug PI3K inhibitor CL27c improves lung function in murine models of glucocorticoid-resistant neutrophilic asthma and fibrosis [24]. In the present study, we further confirmed that inhibition of PI3K by LY294002 attenuated clinical symptoms of neutrophilic airway inflammatory mice. Moreover, in line with these previous studies, LY294002 also significantly reduced inflammatory cells infiltration, especially neutrophils, and mucus production in the airways of our experimental mice [23, 24, 25]. These novel findings further suggest that PI3K signaling pathway may be involved in regulation of Th17-derived neutrophilic airway inflammation [26].

It has been reported that Th17 is the major player in the pathogenesis of mouse and human neutrophilic asthma, in which IL-17 derived from Th17 cells further promotes the recruitment of neutrophils by stimulating the production of neutrophil-attracting cytokines or chemokines from airway epithelial cells [4, 5]. Several studies link PI3K activity with inflammation in allergic airway diseases, and one indicated that rhinovirus infection induced PI3K-dependent neutrophilic airway inflammation [27]. Furthermore, in an OVA-induced asthma model, administration of IC87114, a selective PI3K δ inhibitor, not only attenuated allergic airway inflammation but also significantly reduced the increase in IL-17 protein and mRNA expression [12, 13]. Pharmacologic and genetic blockade of PI3K function restored steroid sensitivity in experimental chronic obstructive pulmonary disease (COPD) [28, 29]. Expectedly, we found that administration of LY294002 significantly decreased the inflammatory cells infiltration in the airways and BALF, and the production of cytokines in BALF. These findings revealed that the provocation of PI3K signaling pathway was critical for airway inflammation. Therefore, we measured the levels of IL-17 and IL-10 in BALF. We found that neutrophil numbers and IL-17 levels were significantly reduced, but IL-10 levels in BALF were significantly increased in BALF in LY294002 treated mice. In line with our study, Liu et al. [30] also found that after ligustrazine treatment, neutrophil numbers and IL-10 levels were significantly increased, whereas that of IL-17 were significantly decreased in BALF. These results showed that there existed imbalance between IL-17 and IL-10 in patients with chronic persistent asthma and it was involved in the development of asthma [31]. Due to increased blood levels of IL-17 and IL-6 were confirmed in patients with severe asthma [3, 8], we further measured the plasma levels of IL-17 and IL-6 in inflammatory mice by ELISA. Similarly, we observed significantly elevated plasma levels of IL-17 and IL-6 in neutrophilic airway inflammatory mice compared to that in control mice, which was in line with previous studies [32]. Moreover, plasma levels of IL-17 and IL-6 were significantly reduced in LY294002 treated mice relative to that in untreated mice, further suggesting that the anti-inflammatory effects of LY294002 on neutrophilic airway inflammation.

Recent studies have shown that the NLRP3 inflammasome was involved in regulation of the neutrophilic airway inflammation in patients with severe asthma [16]. To examine whether the anti-inflammatory role of LY294002 was correlated with the expression of NLRP3 inflammasome, we measured the expression levels of related proteins in challenged mice. In line with previous studies, the significantly increased expression levels of p-Akt, ASC, NLRP3, caspase-1 and IL-1β suggested that the activated PI3K activity and NLRP3 inflammasome in OVA-LPS-induced neutrophilic airway inflammatory mice. Mature IL-1β derived from NLRP3 inflammasome can promote the differential Th17 cells, maintain the production of Th17-associated cytokines, and facilitate allergic inflammation [33, 34]. Studies found that the increased NLRP3, caspase-1 and IL-1β responses drove steroid-resistant neutrophilic inflammation [16]. Moreover, use of specific NLRP3 inflammasome inhibitors reduced HDM-induced AHR and attenuated steroid-resistant asthma in a mouse model [35]. Expectedly, treatment of LY294002 significantly decreased the expression levels of p-Akt, ASC, NLRP3, caspase-1, and IL-1β suggesting that the activated PI3K activity and NLRP3 inflammasome were inhibited by this treatment.

## Conclusions

Overall, our results demonstrate that the PI3K inhibitor LY294002 plays a crucial role in resolving the neutrophilic inflammation through downregulation of the NLRP3 inflammasome in neutrophilic airway inflammatory mice. Thus, the PI3K signaling pathway may provide novel therapeutic approaches for neutrophilic airway diseases.

## Supplementary Information

Below is the link to the electronic supplementary material.

Supplementary file1 (DOCX 264 kb)

## Data Availability

The data used to support the findings of this study are all included within this article.

## Abbreviations

BALF: Bronchoalveolar lavage fluid
Con: Control
Con+LY: Control+LY294002
HE: Hematoxylin eosin
LPS: Lipopolysaccharides
NLRP3: The NOD-like receptor 3
OVA+LPS: Ovalbumin+lipopolysaccharides
OVA+LPS+LY: Ovalbumin+lipopolysaccharides+LY294002
OVA: Ovalbumin
PAS: Periodic acid-Schiff
PI3K: Phosphoinositide 3-kinases
