# Supplementary material for: Inhibition of phosphoinositide 3-kinase activity attenuates neutrophilic airway inflammation and inhibits pyrin domain-containing 3 inflammasome activation in an ovalbumin-lipopolysaccharide-induced asthma murine model
Source: Mol Biol Rep. 2024 May 29;51(1):698. doi: 10.1007/s11033-024-09360-5 (PMC11136729; doi:10.1007/s11033-024-09360-5)
Supplement: Supplementary file 1 — Supplementary file1 (DOCX 264 kb) [file 11033_2024_9360_MOESM1_ESM.docx]

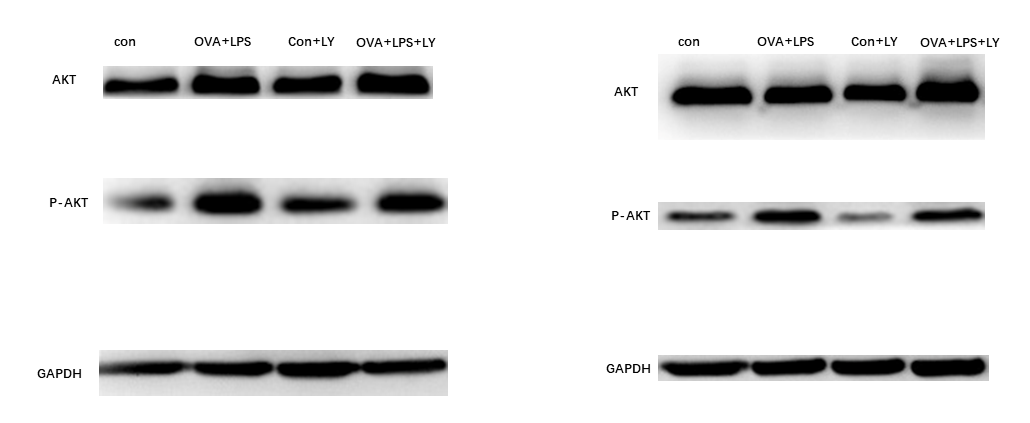


Fig1. After subtracting the blot membrane according to the Mark protein, we then performed antibody incubation.


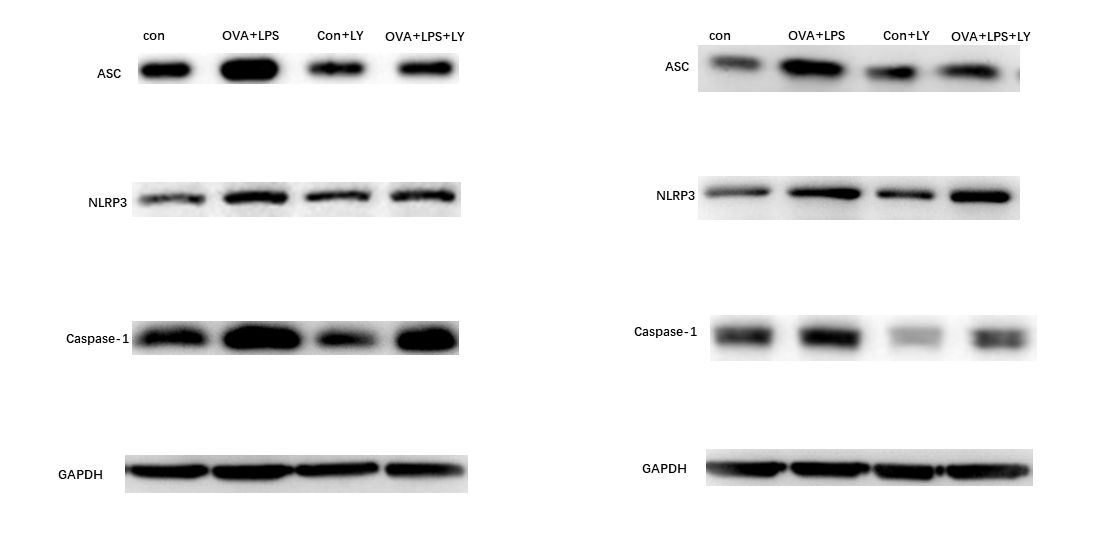


Fig2. After subtracting the blot membrane according to the Mark protein, we then performed antibody incubation.


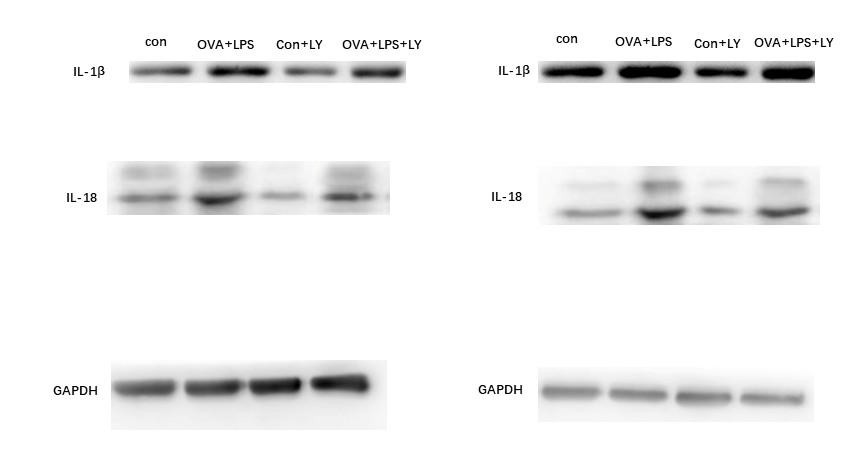


Fig3. After subtracting the blot membrane according to the Mark protein, we then performed antibody incubation.
